# Feeling Unsafe in One’s Own Body: The Impact of Illness on Psychological Safety and Social Engagement

**DOI:** 10.3390/ijerph23020148

**Published:** 2026-01-24

**Authors:** Phoebe Taylor, Liza Morton, Nicola Cogan

**Affiliations:** 1 Department of Psychology, Glasgow Caledonian University, Glasgow G4 0BA, UK; 2 School of Psychological Sciences and Health, University of Strathclyde, Glasgow G1 1XQ, UK; nicola.cogan@strath.ac.uk

**Keywords:** psychological safety, illness experience, polyvagal theory, neuroception, chronic illness, qualitative research, embodiment, social connection

## Abstract

The concept of neuroception of psychological safety, rooted in Polyvagal Theory, offers a framework for understanding how individuals perceive safety at a physiological and psychological level. Illness may disrupt this perception and affect bodily regulation, emotional resilience, social connection, and self-compassion. This study aims to explore how experiences of being unwell, across both acute and chronic contexts, affect individuals’ neuroception of psychological safety. Semi-structured interviews were conducted with eleven adult participants aged 20–79, including individuals with both acute and chronic illness experiences. Interview questions were informed by the Neuroception of Psychological Safety and Polyvagal Theory. Data were analysed using reflexive thematic analysis, following Braun and Clarke’s six-step process. Four key themes were identified: dysregulation and the narrowing window of tolerance (reduced emotional resilience and heightened bodily sensitivity); distrust and disappointment (a rupture in bodily and self-trust); responsibility and internalised guilt (moral and emotional burdens around illness and recovery); and illness demands attention and disrupts social connection (withdrawal, emotional depletion, and compromised compassion). Across these themes, participants described a diminished sense of psychological safety when unwell, shaped by both internal physiological changes and altered social dynamics. Illness can profoundly undermine psychological safety by disrupting neurobiological regulation, altering relational engagement, and eroding trust in one’s body and self. These findings highlight the importance of integrating psychological safety principles into models of care, particularly in how individuals experience and recover from illness.

## 1. Introduction

Individuals living with long-term health conditions are at a significantly increased risk for depression with chronic pain, autoimmune disorders, cancer, heart disease, and diabetes among the most commonly associated conditions [1,2,3]. The relationship between physical illness and mental health is bi-directional: depression can exacerbate physical symptoms, while the burden of physical symptoms can contribute to the onset and worsening of mental health difficulties, including depression and anxiety [4,5]. Suicidality, including suicidal ideation and attempts, is also more prevalent among individuals with chronic or long-term illnesses, with research indicating that the risk of suicidal ideation may be up to three times higher than in individuals without such conditions [6,7]. Factors contributing to this heightened risk include physical pain, functional limitations, emotional distress, and social isolation [8]. Many individuals report a diminished sense of autonomy and self-worth, with illness-related changes to physical appearance or function affecting their body image, self-esteem, and relationships [9,10].

This study is grounded in Polyvagal Theory [11,12,13] and informed by the Neuroception of Psychological Safety Scale (NPSS) [14]. According to Polyvagal Theory, the autonomic nervous system continuously evaluates the environment for cues of safety or threat through a process termed neuroception. When safety is neuroceptively detected, individuals can access the social engagement system, enabling connection, regulation, and calm. Conversely, threat detection initiates defensive physiological strategies such as fight, flight, or shutdown, resulting in increased arousal, muscular tension, and emotional dysregulation [15]. While the concept of psychological safety has gained prominence in organisational and clinical settings, its specific relevance to illness experience, particularly from the perspective of lived experience, remains underexplored. Recent work by Roche et al. [16] highlights the significance of psychological safety during medical procedures for individuals diagnosed with chronic illness in childhood. The study identifies key facilitators of psychological safety in healthcare settings, including knowledge empowerment, family involvement, and holistic care. Their findings emphasise that psychological safety is essential not only for immediate clinical outcomes, but also for long-term well-being [13]. However, the scope of that study focused specifically on early diagnosed chronic illness in medical contexts. Broader questions remain about how psychological safety is experienced across everyday illness contexts, including both acute and chronic conditions, and beyond formal medical encounters [17,18,19,20,21].

Emerging evidence suggests that people with chronic illness may begin to perceive their own bodies as unsafe or unreliable, fostering internalised distress and mistrust [17]. Furthermore, acute illness episodes can also be deeply disruptive, temporarily altering one’s capacity to function, engage socially, or maintain responsibilities [22]. Physical symptoms such as pain, fatigue, or sensory dysregulation may disrupt one’s relationship with their body, fostering feelings of frustration, dependency, or alienation [23,24]. These disruptions extend beyond somatic symptoms to affect emotional and social dimensions of everyday life. Illness can also reshape interpersonal dynamics. The emotional toll of illness can diminish individuals’ capacity to connect with others or offer empathy, further challenging social engagement and relational identity [24,25]. Receiving care, though potentially supportive, may evoke discomfort, role strain, or vulnerability, particularly for those accustomed to caregiving roles [24,25,26].

Theoretical models, such as the Illness Constellation Model [27] and the Trajectory Framework [28], emphasise the multidimensional nature of illness. They explore how illness affects self-identity, interpersonal relationships, and social roles. Polyvagal Theory further asserts that illness may distort the interpretation of environmental cues, potentially impeding emotional regulation and interpersonal connection [12].

This study addresses a gap in the literature by exploring how illness, particularly acute and episodic illness, disrupts neuroception and the felt sense of psychological safety. Drawing on Polyvagal Theory and the NPSS, this study examines how being unwell affects individuals’ sense of bodily trust, emotional regulation, and social connection.

Specifically, the aim of this study is to explore how the experience of being unwell affects individuals’ neuroception of psychological safety. The research examines how illness shapes the embodied sense of safety in relation to three domains: bodily sensations, social engagement, and self-compassion. It also considers how psychological (un)safety, in turn, may influence individuals’ experiences of illness, including how they relate to their bodies and interact with others during times of ill health. The primary research question guiding this study is: How does illness, and the experience of being unwell, affect the neuroception of psychological safety?

## 2. Materials and Methods

### 2.1. Study Design

A qualitative approach was selected to elicit rich, detailed accounts of participants’ personal experiences of being unwell, including those with both acute and chronic health conditions. This approach was well suited to capture the subjective, embodied, and relational aspects of psychological safety, enabling a nuanced understanding of how illness influences one’s felt sense of safety. The study prioritised participants’ lived experiences with a focus on three interrelated domains central to psychological safety: bodily sensations, social engagement, and compassion [14]. By exploring a range of illness experiences, the research sought to identify common patterns and meaningful variations in how individuals navigated disruptions to safety, connection, and self-regulation while unwell. Informed by guidance from Namey et al. [29] who highlight that focused, smaller samples are effective in qualitative research when participants share common, lived experiences, this study initially aimed to recruit approximately ten participants to allow for an in-depth qualitative analysis while ensuring a range of illness experiences. This size was deemed appropriate to allow for in-depth exploration of individual perspectives while maintaining a manageable volume of data for rigorous analysis. Additionally, a smaller sample ensured that the unique voices of participants could be closely examined and accurately represented, aligning with the study’s aim to amplify patient experiences.

### 2.2. Recruitment and Procedure

Participants were recruited through convenience sampling. Eligibility criteria required individuals to be aged 18 or older and willing to reflect on their experiences of being unwell. Both acute and chronic illness experiences were included to capture a broad range of health narratives with no exclusions based on illness type or duration.

Participants were recruited via a digital poster shared through the researchers’ personal and academic networks on social media. Interested individuals contacted the research team via email to express interest. In response, they were sent a participant information sheet outlining the purpose of the study, ethical safeguards, and procedures for participation and withdrawal, along with a consent form. Participants had the opportunity to ask questions before providing written informed consent via email. Semi-structured interviews were conducted remotely via Microsoft Teams. To ensure secure and high-quality audio was captured, a digital dictaphone was used to record each interview.

Two researchers conducted the interviews as part of a joint qualitative study. Each researcher recruited six participants and carried out interviews using a shared interview schedule, which comprised two sections aligned with the respective research aims. After transcription, each researcher retained and analysed the data relevant to their own research questions, while securely sharing the remaining anonymised transcript sections and demographic information with the other researcher. The present analysis and reporting focus solely on the data relevant to the aims of this study.

Interviews lasted up to one hour and followed a participant-centred approach to build rapport and encourage reflection. Informed by the NPSS, the interview schedule focused on three core domains: bodily sensations, social engagement, and compassion [14]. Six open-ended questions guided the interviews, exploring participants’ experiences of illness, its functional and emotional impact, and their perceptions of support and care (see Table 1). Prompts were used flexibly to elicit deeper insight and clarify responses. Interviews began with a broad, grounding question inviting participants to recall a time they had felt unwell, which helped contextualise their reflections within lived experience. Following the interviews, participants were emailed with a debrief form reiterating the study’s aims and providing contact information for support services. Audio recordings were transcribed verbatim by the primary researcher, and all identifying information was removed during transcription. Data were stored securely in accordance with university policy.

All interviews were transcribed verbatim by the first author. To protect anonymity and ensure ethical integrity, all identifying information was removed during transcription. P7’s data was incomplete due to incorrectly followed interview protocol and therefore were excluded from analysis. Data were analysed using reflexive thematic analysis [30,31,32,33], which was particularly well suited to exploring lived experience and participant meaning-making.

Although the interview schedule was informed by Polyvagal Theory and the Neuroception of Psychological Safety framework, the analysis followed an inductive approach within the reflexive thematic analysis tradition. This meant the analytic process remained open to unanticipated meanings, patterns, and insights grounded in participants’ own accounts. Themes were not generated deductively from theory, but developed through a process of active, interpretative engagement with the data [34,35,36].

Reflexive thematic analysis positioned theme development as a creative and interpretive act, shaped by the researcher’s subjectivity, epistemological positioning, and theoretical lens. Rather than seeking consensus coding, reliability testing, or saturation, RTA valued the generation of rich, nuanced interpretations through reflexive meaning-making [31,32,33]. The first phase of analysis involved familiarisation with the data through repeated readings of each transcript. Reflective notes and early impressions were recorded to capture emergent ideas. This informed a process of detailed, line-by-line coding, attending to both semantic (explicit) and latent (implicit) content. Codes were organised using an Excel spreadsheet to support comparison across transcripts and aid in identifying shared patterns.

Theme development was iterative and recursive. As coding progressed, conceptually related codes were grouped into candidate themes. These were then reviewed and refined through constant comparison with attention to internal coherence and thematic distinctiveness. Thematic development was reflexive and dialogic, drawing on the researchers’ positionalities and theoretical sensitivities, while remaining grounded in participants’ lived experiences. To ensure transparency and analytic rigour, a clinical audit trail was maintained throughout the process [34]. This included documentation of coding decisions, thematic development, analytic memos, and reflexive journaling. These records supported traceability from raw data to final themes and enabled collaborative review among the research team. While the first author led the coding, key excerpts and thematic interpretations were reviewed by the second and third authors. Discrepancies and interpretive questions were discussed in depth, allowing for multiple perspectives to enrich the analysis. The final thematic structure was reviewed against the full dataset to ensure that it provided a coherent, meaningful, and credible account of how individuals experience psychological safety in the context of illness.

The data presented in this study are not publicly available due to ethical restrictions and the need to protect participant confidentiality. Anonymised excerpts may be made available upon reasonable request from the corresponding author, subject to ethical approval.

### 2.3. Reflexive Statement

The primary investigator of this study has experienced disability and chronic pain spanning over eight years which shaped the motivation and methodological orientation of this research. These experiences have influenced their understanding of how illness can disrupt bodily trust, emotional regulation, social engagement, and one’s overall sense of psychological safety. Living with persistent health challenges has not only informed the research questions, but also deepened empathy and insight during data collection and analysis. Throughout the process, they have remained reflexively aware of the need to balance experiential sensitivity with analytical rigour to ensure their interpretations were grounded in participants’ narratives, rather than their own assumptions [36].

The second author and primary supervisor also lives with a long-term chronic health condition. Their professional background and personal experience added another important dimension to the analysis and interpretations made. This shared understanding of chronic illness informed collaborative discussions and allowed for a nuanced and compassionate engagement with the data. It also reinforced a core value underpinning this study: that the voices and lived experiences of people affected by illness must be central to any research that aims to understand their needs.

While the third author has not experienced chronic illness personally, they brought valuable indirect experience through a close family member who lives with a long-term health condition. This proximity has given them insight into the social and relational impact of illness on families, including how caregiving roles and emotional dynamics are affected. Their perspective contributed to a broader understanding of how illness is not only an individual experience, but one that reverberates through families and wider social networks. Together, these different yet complementary perspectives enriched the reflexive space of the project. Our team’s positionality enhanced our sensitivity to the data, helped surface important themes, and shaped our shared commitment to research that centres compassion, psychological safety, and embodied understanding in both theory and practice [36].

## 3. Results

### 3.1. Participants

As interviews progressed, it became evident by the eighth interview that the data provided sufficient richness and interpretive depth to support meaningful theme development. Recruitment continued to eleven participants to enhance the breadth and variation in perspectives, and to further support the development and consolidation of the thematic framework.

Participants ranged in age from 20 to 79 years (M = 40.8, SD = 18.3). The sample was predominantly female (*n* = 10; 91%) with one male participant (9%). While not all participants reported a formally diagnosed or long-term health condition, all had experienced significant periods of being unwell, whether acute, episodic, or undiagnosed, that formed the basis for their reflections. These experiences included conditions such as hemiplegic migraine with unpredictable flare-ups lasting days to weeks; undiagnosed pain that persisted for over 18 months, causing daily discomfort and functional limitations; and mild asthma, experienced for over a decade, which affected confidence in physical exertion and prompted heightened bodily vigilance. Others described recurrent fatigue or post-viral symptoms that lasted several weeks and disrupted their work, caregiving responsibilities, and emotional well-being. Regardless of their diagnoses, participants spoke of the cumulative impact of these experiences on their sense of bodily trust, emotional regulation, and capacity for social connection. Most participants lived with others (typically partners and/or children), while one lived alone. In terms of employment, six participants worked part-time, four worked full-time, and one was retired. The majority held roles in organisational, managerial, or caregiving professions, including within health and social care (see Table 2).

### 3.2. Results

Reflexive thematic analysis generated four interconnected themes that captured how the experience of illness impacts psychological safety. The themes reflected disruptions in bodily regulation, trust in self, social connection, and self-compassion.

#### 3.2.1. Dysregulation and Narrowing Window of Tolerance

Participants described how illness compromised their ability to emotionally and physically self-regulate. They reported being more irritable, reactive, or emotionally fragile than usual. Everyday stressors that they would typically navigate with ease became overwhelming, suggesting a narrowing of their window of tolerance, the optimal zone of arousal within which individuals feel psychologically safe and able to adaptively respond.

“*My brain couldn’t cope with what was going on and it led me to be very short-tempered and angry.*”

(P8)

“*Not maybe dealing with situations as well as you would’ve done previously.*”

(P11)

This dysregulation manifested through physiological symptoms, such as nausea, crying, and breathlessness, which were experienced alongside feelings of stress, panic, and emotional shutdown. Participants described a heightened awareness of bodily sensations that contributed to a loss of internal stability.

“*I was really, really stressed… I felt very overwhelmed and there was often a lot of crying.*”

(P8)

“*Anxiety, nausea too, so I can say I didn’t feel safe in my body at all.*”

(P9)

The loss of clarity around bodily cues, especially in the absence of a clear diagnosis, amplified uncertainty and hypervigilance, making participants more sensitive to internal changes and less able to interpret symptoms with confidence.

“*More conscious of all of my physical sensations because of that uncertainty around the diagnosis.*”

(P10)

In line with Polyvagal Theory, participants appeared to shift out of the ventral vagal (social engagement) system and into states of threat and protection. These states limited their access to calm and relational presence, and reduced their felt sense of safety both within their bodies and in the external world. 

#### 3.2.2. Distrust and Disappointment

Illness also disrupted participants’ sense of bodily integrity and trust. Where the body had once been a reliable source of action and strength, it now felt foreign, unpredictable, or fragile. This disruption was emotionally charged, often described in terms of frustration, disappointment, and betrayal.

“*I couldn’t… I couldn’t trust my body. My body, I feel, had let me down.*”

(P9)

“*I felt quite frustrated… quite let down by my body. Like it wasn’t doing what it should be doing.*”

(P10)

For some, this breakdown in trust extended beyond the body to the self more broadly, indicating a deeper rupture in self-efficacy and internal coherence.

“*I just felt so untrusting of myself.*”

(P9)

This theme reflected a central tenet of psychological safety: the ability to feel at home in one’s body. When the body becomes a source of distress or unreliability, the foundational sense of safety and self-regulation is compromised.

#### 3.2.3. Responsibility and Internalised Guilt

Participants often internalised a sense of blame or responsibility for becoming unwell. Some attributed their illness to personal failure, poor self-care, or unresolved stress, reflecting wider societal narratives that moralise health.

“*There’s also guilt there… because I’m not looking after my body enough for this to happen.*”

(P9)

This theme was particularly evident in relation to work. Several participants reported pressure to minimise their symptoms, return to work prematurely, or downplay their distress in order to meet professional expectations. When they were unable to do so, they experienced guilt, shame, and self-criticism.

“*Work didn’t understand that my sports injury had had such a huge impact and I was generally struggling with my mental health at that time.*”

(P12)

“*Exasperated by having a boss who phoned me twice, sometimes three times a week, to see where I was at and when I’d be back.*”

(P10)

The inability to perform or support others, including as a parent, employee, or partner, was deeply distressing for many participants. These internalised expectations intensified the psychological burden of illness and inhibited self-compassion.

“*When I can’t work, I feel extremely guilty because I feel like I’m letting other people down… guilty on others, but also guilty on myself.*”

(P9)

#### 3.2.4. Illness Demands Attention and Disrupts Social Connection

Illness demanded participants’ full physical, emotional, and cognitive attention. Symptoms such as pain and fatigue monopolised their focus, leaving little energy or space for social connection, responsibilities, or self-care.

“*I’d be travelling home more and seeing friends more if I weren’t in this condition.*”

(P8)

“*I spend a lot of time focusing on my own pain, which doesn’t actually help… but it’s hard not to think about it all the time.*”

(P6)

Participants reported withdrawing from relationships and social settings, often not out of apathy but as a strategy to conserve energy or avoid having to explain their condition. For many, the effort of engaging with others was overwhelming.

“*I didn’t want to socialise with people. I didn’t want to explain what was going on.*”

(P10)

“*Even a one-minute conversation felt like too much for my body.*”

(P9)

In addition to reduced social engagement, participants expressed a diminished capacity for empathy. The emotional toll of illness meant that they struggled to extend compassion toward others or maintain caregiving roles they had previously valued.

“*Yeah, I probably cared much less about others.*”

(P12)

“*I still like to support people… but sometimes my brain can’t focus enough to be able to do all these things.*”

(P11)

Solitude was often preferred over interaction, especially during moments of emotional or physical exhaustion.

“*I ended up pushing him away. I wanted him to leave me alone. I just wanted to kind of lie there by myself.*”

(P9)

“*I felt my capacity for any sort of social interaction was zero. I just wanted to be left alone.*”

(P9)

Several participants were also acutely aware of how their illness was affecting others in their life, further contributing to the emotional strain.

“*It’s impacting all my life and all my family’s life… that definitely can make you feel insecure and unsafe and, yeah, worried.*”

(P11)

This theme highlighted how the neuroception of psychological safety can be undermined not only internally, but also relationally, as illness interferes with social reciprocity, compassion, and co-regulation (see Table 3). 

## 4. Discussion

The findings have shown that illness significantly compromises an individual’s ability to remain within their optimal window of tolerance, an internal state where emotional regulation, relational engagement, and cognitive functioning are most accessible [37]. Participants described experiencing heightened physiological arousal, increased emotional sensitivity, and reduced resilience to everyday stressors. These responses were consistent with Polyvagal Theory, which posits that the autonomic nervous system, when detecting threat, shifts individuals into protective states such as fight, flight, or shutdown, inhibiting their capacity for social engagement and co-regulation [11].

The second major theme was the erosion of bodily trust. Many participants experienced their body as unreliable, foreign, or betraying, particularly when symptoms persisted or diagnoses were unclear. This rupture in embodied security undermined their ability to feel safe internally. Prior research similarly suggested that chronic or unpredictable health issues can disrupt body identity and self-coherence, contributing to anxiety, alienation, and distress [38]. The loss of bodily predictability, therefore, emerged as a central threat to psychological safety during illness, whereby participants reported feeling chronically unsafe in their own body [12].

The theme of internalised responsibility further revealed how individuals often blamed themselves for their illness or for failing to recover “appropriately.” Participants reported guilt and shame when they were unable to meet expectations, either their own or others, particularly in work or caregiving contexts. This reflected societal discourses that equate health with self-discipline and illness with failure [38,39]. These beliefs may have inhibited self-compassion and prevented individuals from seeking support, thereby compounding distress and deepening the experience of psychological unsafety. It is perhaps of interest to note that most participants were females, with many employed in health and social care roles. As such, being on the receiving end of care, contrasting with their perceived social responsibilities, which may have evoked discomfort, role strain, or vulnerability [38,39].

The final theme highlighted how illness depletes emotional and cognitive resources, reducing individuals’ capacity to connect with others or offer compassion. Participants reported withdrawing from relationships, avoiding social contact, and struggling to engage empathetically with others’ needs. While these behaviours may have served as adaptive coping strategies, they simultaneously diminished opportunities for co-regulation and relational safety [40,41]. The absence of supportive, attuned connection further compromised recovery and reinforced the isolation often experienced during illness [42]. Collectively, these findings demonstrated that psychological safety is a deeply embodied and relational phenomenon. It is not only disrupted by external threats, but also by internal states such as bodily discomfort, emotional dysregulation, and loss of trust in the self. The study suggested that illness, beyond its physical effects, can destabilise core psychological processes related to safety, connection, and self-regulation. This calls for a broader conceptualisation of healthcare that includes psychological safety as a fundamental dimension of person-centred and psychologically informed care. 

## 5. Strengths and Limitations

A major strength of this study lies in its original focus and theoretical integration. By applying Polyvagal Theory and the NPSS framework to illness experiences, the study can offer a novel lens through which to understand the embodied and psychological impact of being unwell. The use of reflexive thematic analysis enabled rich, nuanced insights into the lived experiences of participants, grounded in their narratives. The inclusion of both acute and chronic illness experiences allowed for a broad exploration of how psychological safety may be affected across different health trajectories. These findings can contribute to a growing body of work on trauma-informed and relational approaches to health and well-being, adding depth to existing understandings of emotional and social responses to illness.

However, the study has several limitations. The sample size was small (*n* = 11), and participants were predominantly female, limiting the generalisability of these findings. Furthermore, the majority of participants were working in health or social care roles which may have broadened their understanding of the healthcare literature. As stated, the study sample was predominantly female and included a high proportion of health professionals, which limits the transferability of the findings to other populations. These characteristics can shape how illness is experienced, interpreted, and narrated. However, this specificity also enabled in-depth exploration of illness experiences within a group characterised by high healthcare literacy and professional caregiving identities. The absence of data on ethnicity, race, and cultural background also precluded intersectional analysis. As with all qualitative research, the findings should be understood as contextually situated rather than representative of the general population. As such, the study reflected a relatively narrow demographic perspective. The study relied on retrospective accounts of illness, and the recency of experiences was not systematically recorded. As memory is shaped by current affective states and interpretive processes [43], recollections may be influenced by emotional framing or narrative reconstruction. Nonetheless, these subjective accounts can offer valuable insight into how illness is processed and remembered. Future research could extend this work by exploring illness experiences in more diverse and gender-balanced samples, including participants from non-health professional backgrounds, to examine how illness is understood and negotiated across different social and occupational contexts.

Finally, although a second researcher reviewed transcript excerpts and thematic structure for consistency, formal inter-rater reliability testing was not undertaken. While reflexive thematic analysis does not require consensus coding, the absence of multiple coders may raise concerns about interpretive bias and analytical rigour. Future studies should aim to recruit more diverse samples, include real-time or longitudinal designs, and consider integrating NPSS scores or physiological measures to triangulate self-reported data and better understand fluctuations in psychological safety.

## 6. Implications

The findings of this study have clear implications for healthcare practice, mental health support, and future research. They suggest psychological safety, particularly in the context of illnesses, is shaped by disruptions in emotional regulation, bodily trust, self-perception, and relational connection. These are not just peripheral concerns, they are fundamental to how individuals experience, cope with, and recover from being unwell. A felt sense of safety can influence not only emotional well-being, but also physical recovery and engagement with care. Healthcare professionals should be trained to recognise signs of psychological unsafety and to consider how their interactions, tone, and expectations may inadvertently reinforce shame, helplessness, or disconnection [44,45]. Psychologically informed and polyvagal-informed approaches could offer frameworks to cultivate environments in which patients feel seen, heard, and regulated in conditions that support healing. Psychological safety should be considered a core dimension of patient-centred care, especially in contexts involving vulnerability, distress, or chronic health challenges [45].

The NPSS offers promise as a tool to help identify patients who may be struggling with embodied or relational safety [14]. When used alongside conventional clinical assessments, it may help inform care plans that respond to psychological as well as physical needs. Interventions such as compassion-focused therapy, body-based regulation practices, peer support programmes, and relational repair work may be particularly effective in restoring safety and self-trust. Future studies should evaluate the effectiveness of these approaches within both acute and chronic illness populations. 

## 7. Conclusions

Illness has the potential to profoundly disrupt psychological safety by compromising internal regulation, diminishing bodily trust, and straining connections with others. This study provides initial qualitative evidence that being unwell is not merely a physical experience, but an embodied, emotional, and relational disruption that shapes how individuals feel within themselves and in relation to their environments. These findings call for a shift in healthcare systems away from models that focus solely on symptoms and towards approaches that recognise psychological safety as a fundamental component of care. When individuals feel safe in their bodies, relationships, and healthcare interactions, they are better able to regulate distress, engage with support, and participate actively in their own recovery. 

Integrating psychological safety into healthcare design and delivery has the potential to enhance emotional well-being and reduce distress, as well as foster compassionate, psychologically informed, and person-centred care [16,45]. It also supports resilience not just in patients, but across care systems tasked with meeting complex emotional and physical needs. Ultimately, this study highlights the need to view psychological safety not as an abstract or secondary concern, but as a vital and actionable dimension of health. Supporting this felt sense of safety enables individuals to navigate illness with greater dignity, connection, and hope, offering a pathway toward more humane and effective care. 

## Figures and Tables

**Table 1:** Interview Schedule and Sample Questions.

Domain	Focus Area	Sample Question/Prompt
Bodily Sensations	Physical experience of illness	“Can you describe how your body felt during the time you were unwell?”
Functioning	Impact on daily functioning and routine	“How did being unwell affect your ability to carry out your usual activities or responsibilities?”
Emotional Experience	Emotional and psychological impact	“How did being unwell make you feel emotionally or mentally?”
Social Engagement	Connection with others while unwell	“Did you want to be around others while you were unwell? Why or why not?”
Receiving Care	Perceptions of support and understanding	“Did you feel understood or supported by others when you were unwell?”
Compassion	Compassion towards self and others during illness	“Were you able to be kind to yourself during this time? What about toward others?”

**Table 2:** Participant Demographic Characteristics.

Participant	Age	Gender	Occupation	Employment Status	Living Situation
P1	22	Female	Auxiliary Nurse	Full-time	With partner
P2	56	Female	Hairdresser	Part-time	With husband and daughter
P3	23	Female	Civil Servant	Part-time	With parents
P4	53	Female	Teacher	Full-time	With husband and children
P5	58	Male	Educator	Full-time	With wife and children
P6	79	Female	Retired	—	Alone
P7 *	27	Female	Trainee Psychologist	Part-time	With others
P8	30	Female	Youth Worker	Full-time	With husband
P9	20	Female	Support Worker	Part-time	With partner
P10	42	Female	Inspector	Part-time	With others
P11	48	Female	Project Manager	Full-time	With husband and children
P12	32	Female	Logistics Coordinator	Part-time	With family

Note: * Participant P7 was excluded from the final analysis due to incomplete data.

**Table 3:** Summary of Themes.

Theme	Description
Dysregulation and the Narrowing Window of Tolerance	Illness reduced participants’ ability to regulate emotional and physiological states. Stressors became more overwhelming and bodily symptoms heightened emotional reactivity, reflecting a shift away from neuroceptive safety.
Distrust and Disappointment in the Body	Participants described a breakdown in trust toward their bodies. The body became a source of perceived threat rather than safety, leading to feelings of frustration, betrayal, and internal disconnection.
Internalised Responsibility and Guilt	Many participants felt guilt or shame related to their illness, often blaming themselves for becoming unwell or for not recovering quickly. These feelings were particularly pronounced in relation to work and caregiving roles.
Illness Demands Attention and Disrupts Social Connection	The experience of illness absorbed emotional and cognitive resources, limiting capacity for social engagement and compassion. Participants often withdrew from others, leading to increased isolation and reduced opportunities for co-regulation.

## Data Availability

The data presented in this study are not publicly available due to ethical restrictions and the need to protect participant confidentiality. Anonymised excerpts may be made available upon reasonable request from the corresponding author, subject to ethical approval.
